# Incidence and risk factors for surgical site infection (SSI) after primary hip hemiarthroplasty: an analysis of the ACS-NSQIP hip fracture procedure targeted database

**DOI:** 10.1186/s42836-022-00155-2

**Published:** 2023-01-03

**Authors:** Arjun Gupta, John Shin, Dylan Oliver, Michael Vives, Sheldon Lin

**Affiliations:** Department of Orthopaedic Surgery, Rutgers New Jersey Medical School, 140 Bergen Street, Suite D-1610, Newark, NJ 07103 USA

**Keywords:** Hip hemiarthroplasty, Surgical site infection, Geriatric, Trauma, Femoral neck fracture, NSQIP

## Abstract

**Introduction:**

Primary hip hemiarthroplasty (HHA) is frequently utilized to treat geriatric hip fractures, which are associated with significantly higher morbidity and mortality. While not particularly common, surgical site infection (SSI) is a major complication that frequently requires revision surgery in a frail population. The objective of this study was to determine the incidence of and risk factors for SSI after HHA in hip fracture patients.

**Materials and methods:**

This retrospective cohort study was performed using the American College of Surgeons (ACS) National Surgical Quality Improvement Program (NSQIP) database. Geriatric patients (65+) who underwent HHA for non-pathologic, traumatic hip fractures between 2016–2017 were included. Demographic variables, comorbidities, operative variables, and complications were compared between "SSI" and "non-SSI" groups. Multivariate regression identified independent risk factors for postoperative SSI. Significance was set at *P* = 0.05.

**Results:**

A total of 6169 patients were included. The overall incidence of SSI was 1.3%. SSI was significantly associated with body mass index (BMI), preoperative functional status, congestive heart failure, chronic corticosteroid use, intraoperative time, sepsis, wound dehiscence, readmission within 30-days, and reoperation. On multivariate analysis, chronic steroid use (OR: 2.30, 95% CI: 1.13–4.70), BMI ≥ 35 kg/m^2^ (OR: 3.59, 95% CI: 1.57–8.18), and intraoperative time ≥120 mins (OR: 2.15, 95% CI: 1.08–4.27) were found to be independent risk factors.

**Conclusions:**

Postoperative SSI is a serious complication that is responsible for prolonged hospital stays, increased mortality, and greater healthcare costs. Here, we identified multiple risk factors for SSI after primary HHA in the US elderly population.

## Introduction

Geriatric hip fractures are common traumatic injuries encountered by orthopedic surgeons [1]. The incidence of hip fractures continues to grow partially due to the aging of global populations and an increasing frequency of comorbidities. Certain projections estimate more than 700,000 cases will appear annually by 2050, at a net cost of $15 billion in the United States alone [2, 3]. Current trends suggest that primary hip hemiarthroplasty (HHA) is the procedure of choice for repair of displaced femoral neck fractures in elderly patients [4]. However, HHA has been associated with a higher rate of complications compared to elective total hip arthroplasty (THA), and morbidity and mortality in geriatric hip fractures remain high [1, 5].

Infections comprise a substantial fraction of postoperative complications following HHA [1]. In fact, numerous studies have investigated the incidence and underlying risk factors for infections after hip fracture surgery, including pneumonia, urinary tract infections (UTIs), sepsis, and *C. difficile* colitis [6–8]. Surgical site infection (SSI) is another major infectious complication commonly seen after hemiarthroplasty and is responsible for prolonged hospital stays, frequent readmissions, increased mortality, and greater healthcare costs [9]. SSI can cause serious damage to the repaired joint and surrounding soft tissues, often compromising the arthroplasty and requiring revision surgery. This is of particular concern among the elderly. The prevalence of concomitant diseases that are associated with infection risk (*i.e*., diabetes mellitus, atherosclerotic peripheral vascular disease, malignancies) commonly increases with age [10, 11]. The increased incidence of cancer among elderly persons also has implications for infection risk with respect to immunoablative chemotherapy or radiation therapy. Additionally, the elderly population is disproportionately affected by inadequate nutritional intake—another correlate of infection risk—perhaps due to the diminished digestive and metabolic functions associated with senescence [12]. Limited mobility, protracted recovery, pathogen exposure in hospitals or long-term care facilities, challenges with self-care, and delayed wound healing may also play a contributory role [13, 14]. However, reported SSI rates after HHA have been highly variable, with estimates ranging between 1.6–10% [15–18]. Additionally, previous studies aiming to identify the modifiable risk factors for SSI have been limited by small sample sizes, clumping of distinctly different procedures (*e*.*g*., closed reduction percutaneous pinning, open reduction internal fixations, intramedullary fixations, THA, and revision surgeries), and subjects of non-US populations [7, 15, 16, 18, 19]. To our knowledge, no report of the predictive factors of SSI after primary HHA in the United States has been published in the literature to date.

The objective of this study was to utilize data from the American College of Surgeons (ACS) National Surgical Quality Improvement Program (NSQIP) to determine the incidence of SSI and categorically describe the risk factors for SSI after HHA in geriatric hip fracture patients. Given the increasing utilization of primary HHA and the persistently high rate of morbidity and mortality after femoral neck fractures, understanding the correlates of postoperative SSI can aid in mitigation of risk and proactive management of vulnerable patients [20].

## Materials and methods

### Data source

The American College of Surgeons (ACS) National Surgical Quality Improvement Program (NSQIP) database and NSQIP Hip Fracture Targeted Procedure Dataset were utilized for the present study. The NSQIP initiative began through the Veteran’s Administration (VA) health system as a quality improvement program in 1994. Its efficacious implementation in the VA health system led to a parallel program for private hospitals in 1998. For the year 2017, the most recent year included in this study, the NSQIP database contained over one million cases collected from approximately 700 hospitals. Risk-adjustment enables each hospital to make a valid comparison of its outcomes with those of other hospitals in order to identify areas for quality improvement.

The NSQIP database prospectively collects over 150 patient-related variables, including but not limited to, demographics, comorbid conditions, and postoperative complications through analysis of medical records, operative reports, and patient interviews. The NSQIP hip fracture targeted procedure dataset utilized herein additionally collects 16 variables specific to hip fracture cases. Data are collected prospectively by clinically-trained personnel using a standardized entry form, and database quality is maintained by on-site surgical clinical reviewers and the NSQIP's internal auditing process, which improves inter-rater reliability and ensure accuracy of data collection. Specific definitions and diagnostic criteria for each variable/complication can be found in the ACS Quality Improvement Program electronic operations manual (http://cqi.facs.org/operationsmanual/mbsaqip/Chapters_and_Appendices/Chapter_4.htm).

### Study population

The NSQIP databases for 2016 and 2017 were queried. Patients that underwent hip hemiarthroplasty were identified using Current Procedural Terminology (CPT) code 27236. For the selection of typical geriatric hip fracture cases, patients under the age of 65 were excluded. This cutoff age was selected based on the World Health Organization's definition of elderly persons as those 65 years and older [21, 22]. Also, patients with hip fracture due to pathologic etiology and those undergoing concomitant interventions were excluded. Those with American Society of Anesthesiologists (ASA) class 5 or unknown ASA class were excluded. Patients were divided into two cohorts based on the presence or absence of postoperative SSI. Postoperative SSI was defined as any superficial incisional, deep incisional, or organ/space SSI occurring within 30 days after the primary procedure. Superficial incisional SSI was classified as involving only the skin or subcutaneous tissue; deep incisional SSI was classified as involving deep soft tissues, such as fascial and muscle layers; organ/space SSI was classified as involving any part of the anatomy (*e*.*g*., organs or spaces) other than the incision, which was opened or manipulated during the primary procedure. Specific diagnostic criteria for each SSI type can be found in the ACS Quality Improvement Program electronic operations manual (http://cqi.facs.org/operationsmanual/mbsaqip/Chapters_and_Appendices/Chapter_4.htm).

### Patient factors and outcome variables

Demographic factors assessed included age, sex, body mass index (BMI), functional status, and the use of bisphosphonates. Comorbid conditions assessed were congestive heart failure (CHF), hypertension (HTN) requiring medication, current smoking status, dyspnea, chronic obstructive pulmonary disorder (COPD), dialysis, unintentional weight loss (defined by NSQIP as greater than 10% decrease in body weight in the 6 month interval immediately preceding surgery), diabetes, chronic steroid use, preoperative dementia, preoperative delirium, preoperative pressure sore, preoperative transfusion, and bleeding disorders. Operative variables included operative time and ASA class, grouped by classes 1–2 and 3–4.

Postoperative complications analyzed were myocardial infarction (MI), pneumonia, unplanned re-intubation, failure to wean from ventilator > 48 h postoperatively, acute renal failure, progressive renal insufficiency, wound dehiscence, urinary tract infection (UTI), deep venous thrombosis (DVT), pulmonary embolism (PE), intraoperative or postoperative transfusion, readmission, reoperation, and mortality.

### Statistical analysis

Patient demographic variables, comorbid conditions, operative variables, and postoperative complications were compared between those that did and did not develop SSI. Chi-square test was used for categorical variables and Student's *t*-test was used for continuous variables. Variables associated with SSI with *P*-value of less than 0.2 were subsequently controlled in the multivariate analyses, which were performed to identify independent risk factors for SSI. All statistical analyses were performed using SAS software (Version 9.3, SAS Institute Inc., Cary, NC, USA). Statistical significance level was set at *P* = 0.05.

## Results

A total of 6169 geriatric patients (65+) who underwent HHA for femoral neck fracture were identified from the NSQIP database (2016–2017) for inclusion in this study (Table 1). Of these, 80 cases developed postoperative SSI, yielding an overall incidence rate of 1.3%.

**Table 1:** Demographics of geriatric patients undergoing hip hemiarthroplasty stratified by occurrence of surgical site infection

	No SSI	SSI	***P***-value
**Total **(***n***)	5989	80	
**Age (%)**			**0.2650**
65–80	28.39	28.75	
80–90	46.60	53.75	
90+	25.01	17.5	
**Sex (%)**			**0.1572**
Female	68.64	61.25	
Male	31.36	38.75	
**Body mass index (%)**			0.0019*
Underweight (<18.5 kg/m^2^)	20.17	21.25	
Normal weight (18.5–29.9 kg/m^2^)	69.21	56.25	
Overweight (30.0–34.9 kg/m^2^)	7.76	13.75	
Obese (≥35 kg/m^2^)	2.86	8.75	
**Pre-operative functional status (%)**			0.0029*
Independent in ADL	76.29	60.00	
Partially/totally dependent in ADL	23.18	38.75	
Unknown	0.53	1.25	
**Bone medications (%)**			**0.2550**
Yes	30.36	36.25	
No	69.64	63.75	

*Abbreviations*: *SSI* Surgical site infection, *ADL* Activities of daily living

*Statistically significant (*P* < 0.05)

Univariate analysis of patient characteristics identified BMI and preoperative functional status as significant correlates of SSI (*P* = 0.0019 and *P* = 0.0029, respectively) (Table 1). Patient age and use of bisphosphonates were not significantly associated with SSI. Comorbid conditions associated with SSI were congestive heart failure and chronic corticosteroid use (*P* = 0.0359 and *P* = 0.0205, respectively) (Table 2). Preoperative delirium and bleeding disorders were not significantly associated with postoperative SSI (*P* = 0.1482 and *P* = 0.1984, respectively). Increased intraoperative time, but not ASA score, was a significant operative variable associated with SSI (*P* = 0.0219) (Table 3). Development of postoperative SSI correlated with other postoperative complications, including sepsis, wound dehiscence, readmission within 30-days, and reoperation (*P* < 0.0001 for all) (Table 4).

**Table 2:** Comorbid conditions of geriatric patients undergoing hip hemiarthroplasty stratified by occurrence of surgical site infection

	No SSI	SSI	***P***-value
**Total **(***n***)	5989	80	
**Comorbidities (%)**			
Congestive heart failure	4.06	8.75	0.0359*
Hypertension requiring medication	68.28	65.00	0.5319
Current smoker	8.11	5.00	0.3097
Dyspnea	7.50	6.25	0.6736
Chronic obstructive pulmonary disorder	9.95	11.25	0.7002
Currently on dialysis	1.49	0	0.2720
Weight loss, unintentional	1.25	0	0.3139
Diabetes	15.86	13.75	0.6072
Chronic corticosteroid use	5.34	11.25	0.0205*
Pre-operative dementia	33.53	37.50	0.4549
Pre-operative delirium	13.22	18.75	0.1482
Pre-operative pressure sore	3.84	3.75	0.9667
Pre-operative transfusion	2.19	2.50	0.8495
Bleeding disorder	17.05	22.50	0.1984

*Abbreviation*: *SSI* Surgical site infection

*Statistically significant (*P* < 0.05)

**Table 3:** Perioperative variables of geriatrics undergoing hip hemiarthroplasty stratified by occurrence of surgical site infection

	No SSI	SSI	***P***-value
**Total **(***n***)	5989	80	
**Intraoperative time, mins (%)**			0.0219*
0-60	39.86	32.50	
60-90	33.33	40.00	
90-120	18.25	11.25	
> 120	8.57	16.25	
**ASA classification (%)**			**0.2294**
1 or 2	14.79	10.00	
3 or 4	85.21	90.00	

*Abbreviations*: *SSI* Surgical site infection, *ASA* American Society of Anesthesiologists

*Statistically significant (*P* < 0.05)

**Table 4:** 30-day postoperative adverse events in patients undergoing hip hemiarthroplasty stratified by occurrence of surgical site infection

	No SSI	SSI	***P***-value
**Total **(***n***)	5989	80	
**Mortality (%)**	6.48	6.25	0.9342
**Systemic (%)**			
Sepsis	1.27	22.50	< 0.0001*
**Cardiovascular (%)**			
Myocardial infarction	2.57	2.50	0.9680
**Pulmonary (%)**			
Unplanned intubation	1.24	0	0.3171
Pneumonia	4.12	6.25	0.3438
**Renal (%)**			
Acute renal failure	0.33	1.25	0.1657
Progressive renal insufficiency	0.37	0	0.5871
**Hospital-acquired conditions (%)**			
Wound dehiscence	0.08	2.50	< 0.0001*
Urinary tract infection	4.41	5.00	0.7980
Deep venous thrombosis	0.85	1.25	0.7009
Pulmonary embolism	0.82	1.25	0.6712
**Other (%)**			
Transfusion, perioperative	15.61	18.75	0.4429
Readmission	8.40	52.50	< 0.0001*
Reoperation	2.10	48.75	< 0.0001*
**Failed wean (%)**	0.63	1.25	0.4937

*Abbreviation*: *SSI* Surgical site infection

*Statistically significant (*P* < 0.05)

Multivariate analysis identified multiple independent predictors for SSI, including obesity classes 2 or 3 (OR = 3.586, 95% CI = 1.572–8.180, *P* = 0.0261), chronic steroid/immunosuppressant use (OR = 2.304, 95% CI = 1.128–4.704, *P* = 0.0219), and intraoperative time exceeding 120 mins (OR = 2.148, 95% CI = 1.080–4.270, *P* = 0.0211) (Table 5). Chronic steroid/immunosuppressant users were defined as patients who were actively undergoing steroid/immunosuppressant therapy for a chronic condition for at least 10 days cumulatively within the 30 days prior to surgery. Intraoperative time was defined as the elapsed time from initial incision to completion of all procedure-related activities in the operating room. Other factors, including sex, baseline functional status, preoperative delirium, or bleeding disorders were not identified as independent predictors for SSI.

**Table 5:** Multivariate analyses of potential risk factors for surgical site infection after hip hemiarthroplasty in geriatric patients

	Odds Ratio (95% CI)	***P***-value
**Sex**		
Male *vs.* female	1.357 (0.856–2.153)	0.1942
**Body mass index**		
Underweight *vs.* normal weight	1.328 (0.752–2.346)	0.1971
Overweight *vs.* normal weight	2.144 (1.092–4.208)	0.4871
Obese *vs.* normal weight	3.586 (1.572–8.18)	0.0261*
**Pre-operative functional status**		
Partially/totally dependent *vs.* independent	2.214 (1.379–3.553)	0.5465
Unknown *vs.* independent	2.524 (0.327–19.496)	0.6104
**Congestive heart failure**		
Yes *vs.* no	1.787 (0.796–4.01)	0.1595
**Steroid use**		
Yes *vs.* no	2.304 (1.128–4.704)	0.0219*
**Pre-operative delirium**		
Yes *vs.* no	1.226 (0.68–2.209)	0.4981
**Bleeding disorder**		
Yes *vs.* no	1.273 (0.742–2.185)	0.3811
**Intraoperative time, mins**		
60–90 *vs.* 0–60	1.469 (0.868–2.485)	0.3386
90–120 *vs.* 0–60	0.739 (0.343–1.591)	0.0569
> 120 *vs.* 0–60	2.148 (1.08–4.27)	0.0211*

*Abbreviations*: *SSI* Surgical site infection, *95% CI* 95% confidence interval

*Statistically significant (*P* < 0.05)

## Discussion

The mounting incidence of geriatric hip fractures and persistently high postoperative morbidity and mortality rates are cause for concern among orthopedic surgeons. Although primary HHA is currently the most commonly indicated procedure for traumatic, displaced femoral neck fractures, particularly in elderly patients, HHA suffers from a high rate of infectious complications, including pneumonia [1], UTIs [1], *C. difficile* colitis [8], and sepsis [7]. Prior literature suggesting that infections are a major source of postoperative morbidity has spurred a growing effort to investigate the incidence and risk factors for such complications. However, these trends remain poorly studied for postoperative SSI in the context of the US geriatric population, despite reports suggesting that SSI is responsible for prolonged hospital stays, increased mortality, and greater healthcare costs [9]. In this study, we aimed to determine the incidence of SSI and categorically describe the risk factors for SSI after hip hemiarthroplasty using 30-day postoperative reports from the NSQIP database.

Analysis of 6169 geriatric hip fracture patients who underwent HHA from 2016–2017 in the United States revealed an overall SSI rate of 1.3% (Table 1). This statistic was slightly lower than incidences reported by studies conducted in other countries, which documented SSI occurrence in 1.6–10% of all cases [15–18]. Generally, HHA has been associated with a higher risk of complications compared to other hip surgeries, particularly elective procedures such as total hip arthroplasty (THA) [1, 5, 18]. The 90-day postoperative incidence of deep infection after THA was reported to range from 0.2–0.9%, much lower than that of HHA [23].

Multivariate analysis identified three independent risk factors for postoperative SSI in this study cohort: obesity (BMI ≥ 35 kg/m^2^), chronic steroid/immunosuppressant use, and prolonged intraoperative time (Table 5). Numerous studies support the finding that increased BMI correlates with profound risk of SSI after surgical repair of hip fractures, among other complications. Bohl *et al.* found that patients with BMI of 25–30 kg/m^2^ and ≥ 30 kg/m^2^ were more vulnerable to SSI within thirty days of geriatric hip fracture surgery (OR: 1.3 [1.0–1.8] and 2.7 [2.0–3.6], respectively) compared to patients with BMI of < 25 kg/m^2^ (*P* < 0.001) [7]. Similarly, Liu *et al.* reported an elevated risk profile for patients with BMI of > 26.6 kg/m^2^ (OR: 2.97, *P* < 0.001) [19]. A more nuanced trend published by Akinleye *et al.* suggested that while deep SSI risk correlated linearly with increased BMI (*P* < 0.001) as previously reported, superficial wound infection rates were actually lower among both underweight and morbidly obese patients [24].

Similarly, longer operative time is commonly reported to independently increase the risk of postoperative complications including SSI (Table 3) [18, 19]. Interestingly, one prior study highlighted a potential risk with very short intraoperative time (≤45 mins), perhaps owing to extensive tissue handling or lack of adequate hemostasis resulting in postoperative hematoma and risk of infection [15]. However, short intraoperative time was not associated with postoperative SSI in our investigation. This may be attributed to the larger sample size used in the present study (*n* = 6169) compared to the previous study (*n* = 92).

Chronic steroid use has not been previously identified as an independent risk factor, except in the context of "late" SSI (>3 months postoperative) after total and hemiarthroplasty [25]. However, sustained immunosuppression due to steroid therapy would likely render patients vulnerable to infectious complications postoperatively. Overall, preoperative steroid use has been documented as an independent predictor of infection after surgical procedures [26].

Unlike elective THA, hemiarthroplasty is typically performed urgently in the settings of acute hip fractures, particularly in the elderly population. Thus, many of the patient risk factors are non-modifiable in practice. In lieu of preparative lifestyle modifications to mitigate risk, a thorough history taking may help identify patients who are at higher risk of postoperative SSI, including those who are on chronic steroids/immunosuppressants. Additionally, rigorous planning, such as ensuring all surgical equipment is available and ready, familiarizing surgical staff with the equipment, and establishing a dedicated hip fracture team, may help minimize intraoperative time and further reduce risk.

A number of other SSI control measures have been discussed in the orthopedic literature, with ongoing debate regarding the efficacy of certain practices [27]. Generally, these practices can be separated into “standard" procedures that have been extensively adopted and other measures that are used more selectively based on physicians' preference. Standard practices include intraoperative antibiotics, redosing antibiotics intraoperatively, postoperative antibiotic protocol, double gloving, and limiting traffic through the operative room. However, some reported that prophylactic antibiotic administration after wound closure has not demonstrated a significant clinical benefit, instead contributing to antimicrobial resistance after prolonged use [28–31].

Decolonization with chlorhexidine bath or other topical antimicrobials the night before surgery may be additionally indicated in high-risk patients to reduce the risk of MRSA infection [32–35]. Intrawound irrigation with Betadine (povidone-iodine) or other surfactant/detergent has been shown to significantly reduce infection rates [36–38]. Direct intrawound antibiotic application is another frequently deployed measure in high risks patients. In patients receiving cemented prostheses, evidence from the Norwegian Arthroplasty Registry suggests a benefit of using antibiotic-impregnated cement, although this did not improve risk of revision surgery compared to uncemented prostheses [39].

This study comes with several limitations. As a retrospective investigation, the analyses only demonstrated associations between variables and cannot adequately determine causal relationships. Additionally, measures to minimize SSI risk such as betadine irrigation or intrawound antibiotic use were not recorded in the NSQIP database and therefore could not be accounted for. Finally, outcomes reporting in the NSQIP database is limited to a 30-day postoperative period, and the SSI rate that was reported in the current study may be an underestimation as SSIs occurring outside of this window could not be included. Chronic or late SSI taking place more than 30 days after surgery has been documented previously [40]. Nevertheless, the NSQIP is a powerful, well-established database that has been used previously to examine SSI risk factors for other orthopedic procedures [1, 23, 41–43].

Despite limitations of the NSQIP database, the present study provides a thorough assessment of post-HHA SSI incidence and risk factors for geriatric hip fracture patients using a large, US-based patient population. Previous studies have been limited by small sample size and clumping of distinctly different procedures such as THA and revision surgeries. While some studies on the subject have been performed in Europe and Asia, intraoperative and perioperative protocols may differ in other countries, thereby making it challenging to extrapolate results from foreign studies to US patient populations. Here, we reported an overall SSI incidence of 1.3% and identified three major independent predictors of SSI risk: obesity (BMI ≥ 35 kg/m^2^), chronic steroid/immunosuppressant use, and prolonged intraoperative time (≥120 mins). In addition to enhancing the informed consent process, these findings can help orthopedic surgeons identify high-risk patients and proactively implement appropriate SSI control measures. Further research is needed to identify appropriate procedures to reduce SSI risk in the identified populations.

## Conclusions

Postoperative SSI is a serious complication that is responsible for prolonged hospital stays, increased mortality, and greater healthcare costs. In this study, we examined the incidence and risk factors for SSI in geriatric patients undergoing primary HHA for femoral neck fracture. The overall incidence of SSI across 6169 patients was 1.3%. Multivariate regression identified three major independent predictors of SSI risk: obesity (BMI ≥ 35 kg/m^2^), chronic steroid/immunosuppressant use, and prolonged intraoperative time (≥120 mins). Despite limitations of the NSQIP database, the findings in this study benefit from a large, US-based patient population and may help orthopedic surgeons proactively identify patients at high risk for postoperative SSI.

## Data Availability

All data for this study were extracted from the publicly-available NSQIP database.
